# Elucidating the Polymorphism of Xanthone: A Crystallization and Characterization Study

**DOI:** 10.1021/acs.cgd.3c01506

**Published:** 2024-03-28

**Authors:** Janine Andrea Preston, Emmanuele Parisi, Brent Murray, Arwen I. I. Tyler, Elena Simone

**Affiliations:** † School of Chemical and Process Engineering, University of Leeds, Leeds LS2 9JT, United Kingdom; ‡ Department of Applied Science and Technology (DISAT), Politecnico di Torino, 10129 Torino, Italy; § Food Colloids and Bioprocessing Group, School of Food Science and Nutrition, University of Leeds, Leeds LS2 9JT, United Kingdom

## Abstract

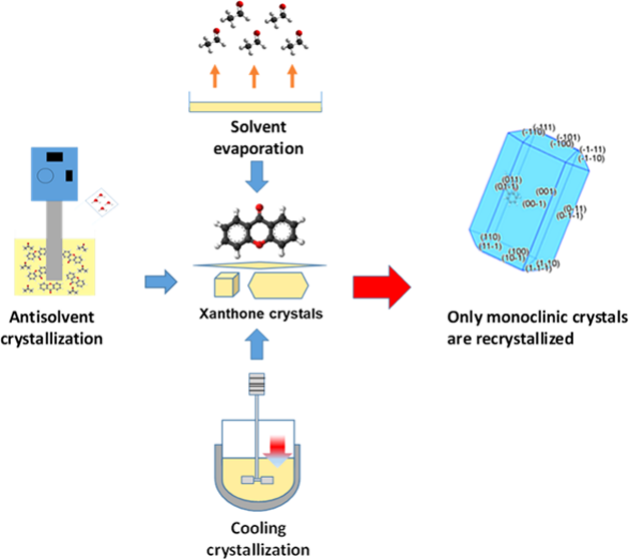

The aim of this work is to shed light on the polymorphism of xanthones, a class of oxygenated molecules well known for their bioactivity, including antioxidant, anticancer, and anti-inflammatory effects. Understanding the polymorphism of xanthones can enable the design of novel solid products for pharmaceutical, nutraceutical, and agrochemical applications. Prior to this work, two entries accounting for different space groups were deposited for 9-xanthone in the Cambridge Structure Database (CSD): an orthorhombic *P*2_1_2_1_2_1_ and a monoclinic *P*2_1_ structure solved at room and low temperatures, respectively. However, the very high similarity between these two structures and the lack of clear differences in their physical properties (e.g., thermal behavior) suggested the possibility of the existence of only one crystal structure. In fact, the differences shown in the literature data might be related to the chosen operating parameters, as well as the instrumental resolution of the single-crystal X-ray diffraction experiments. In the work presented here, the ambiguity in the polymorphism of xanthone is investigated using thermal analysis, powder and synchrotron single-crystal XRD, and optical microscopy. Additionally, a workflow for the correct identification of twinned crystal structures, which can be applied to other polymorphic systems, is presented. Such workflow combines the collection of a large data set of high-resolution diffraction patterns using synchrotron radiation with the use of principal component analysis, a dimensionality reduction technique, for a quick and effective identification of phase transitions happening during the data collection. Crystallization experiments were designed to promote the formation of different crystal structures of xanthone that were recrystallized based on past literature and beyond.

## Abstract 2

Crystallographic and crystallization study on xanthone, combining synchrotron experiments and use of principal component analysis

## Introduction

Interest in the phenomenon of polymorphism has increased markedly in the last two decades, particularly within the pharmaceutical, advanced materials, agrochemical, and even food industries.^1,2^ In foods, for instance, the cocoa butter used as the ingredient in chocolate has six reported polymorphs; each of these polymorphs could exhibit different properties in the final chocolate product (e.g., differences in melting point, sensory/organoleptic properties, thermal stability, and emulsion stability).^3−6^ In organic semiconductors, different polymorphs of fused-ring heteroaromatics could show different HOMO–LUMO bandgaps, electron/hole transport properties, and topochemical reactivity.^7,8^ In pharmaceuticals, polymorph screening is considered an irreplaceable step during the drug development process, since polymorph type could affect the stability, solubility, and bioavailability of pharmaceutical formulations.^9^

Different crystal structures of the same compound can exhibit very different physical and mechanical products. Hence, establishing the solid forms landscape of a compound (including polymorphs, solvates, hydrates, co-crystals) and the transformation pathways between each of these forms is an increasingly important part of development of a new product.^10−13^ Furthermore, tailoring the crystal structure of a compound in order to achieve targeted physicochemical properties is a key challenge for crystal engineers.^14−17^

Solid-form screening protocols explore a wide range of crystallization strategies (e.g., solvent evaporation, cooling crystallization, crystallization from melts carried out in different classes of solvent) at a range of temperatures, pressures, and humidity levels.^18^ Changes in the morphology or the presence of two or more crystal shapes in recrystallized samples are often viewed as indicators of the possible presence of polymorphs, although morphology differences for the same polymorphic form can also be observed when crystals are grown in different solvents.^19^ Solid-form screening can often be a difficult task due to the occurrence of elusive or disappearing crystal structures, which are sometimes difficult to consistently isolate and characterize. Additionally, real crystals present defects, and many also have structural disorder, which makes the structural characterization of these samples very challenging. Beyond our average static view of crystals (averaged positions determined by X-ray diffraction, often at low temperatures), molecules are able to vibrate and change conformation, and in some cases, some of their groups are able to rotate. Furthermore, the quality of the crystals used to solve a structure, the choice between powder and single-crystal sampling, as well as the numerical techniques applied for refinement can influence the reliability of the determined structure.^20−23^ Moreover, different macroscopic phenomena (i.e., mosaicity, disorder, and twinning) and experimental artifacts originating from mishandling of diffraction data can lead to erroneous definitions of new polymorphic crystal structures.^24^

Marsh et al.^25^ reported that the space groups of 60 crystal structures collected from single-crystal measurements had been corrected and assigned to a higher Laue class than what was stated in their original papers. Müller^26^ commented that the assignment of the wrong space group frequently occurs as a result of the assignment of a too low a symmetry or when structures are solved from twinned crystals. Twinning can lead to the presence of pseudosymmetry, which can either be translational or rotational;^27^ pseudosymmetry is a cause of the association of wrong atom coordinates, bond lengths, or interatomic distances in proposed structures. All of this generates uncertainty about the correct space group of a specific solid structure, and in turn, can lead to time and resources consuming solid product development processes. As a practical example, Karami et al.^28^ discussed the reassessment of single-crystal data for the compounds furosemide and finasteride: the authors found that incomplete data collections and systematic weak reflections were the cause of incorrect polymorph identification. Xanthone, the molecule reported in this work, was determined so far in the orthorhombic *P*2_1_2_1_2_1_ space group (CSD ref Code: ZZZTXI01, ZZZTXI02 ZZZTXI011, ZZZTXI12, ZZZTXI13)^29−33^ or as monoclinic, as shown by Trapp^34^ (Form II, CSD ref code ZZZTXI14). The monoclinic structure reported by Trapp et al.^34^ was solved from a twinned crystal, with very similar unit cell and crystal packing to the other known polymorph, but further details on the properties of this polymorph were not provided (e.g., melting point, solubility, biological activity). Saršu̅ns et al.^35^ even questioned the existence of Form II, considering that they were able to crystallize only the orthorhombic Form I and speculated that Form II might result from changes in molecular orientation that could lead to the difference in packing conformation observed between the two polymorphs. They concluded that the orthorhombic form (form I) is the dominant crystalline phase (thermodynamically stable) at room temperature, while other polymorphs could be stable at different temperatures.

Given these premises and considering that the polymorphism of xanthone is still not fully clarified, in this work, we will explore it using single-crystal X-ray diffraction data. Additionally, we developed a useful workflow that could be applied to other crystalline materials whose structural determination is challenging (for example, other twinning crystals). Xanthone is a flavonoid molecule of great interest for food and pharmaceutical formulators due to its antibacterial and anticancer properties.^36−38^ Together with other phenolics, xanthone has also been found to exert a positive effect in the fight against obesity by preventing lipid accumulation in the liver.^39^

In the work presented here, the ambiguity in the polymorphism of xanthone is investigated, and a workflow for the correct identification of twinned crystal structures is presented. Xanthone was recrystallized in several solvents and operating conditions, both based on the previous literature and specifically designed for this study. To determine xanthone polymorphism, crystals were examined via synchrotron single-crystal X-ray diffraction, differential scanning calorimetry, and optical microscopy. Solubility in different solvents was also estimated.

## Materials and Methods

**Figure 1:**
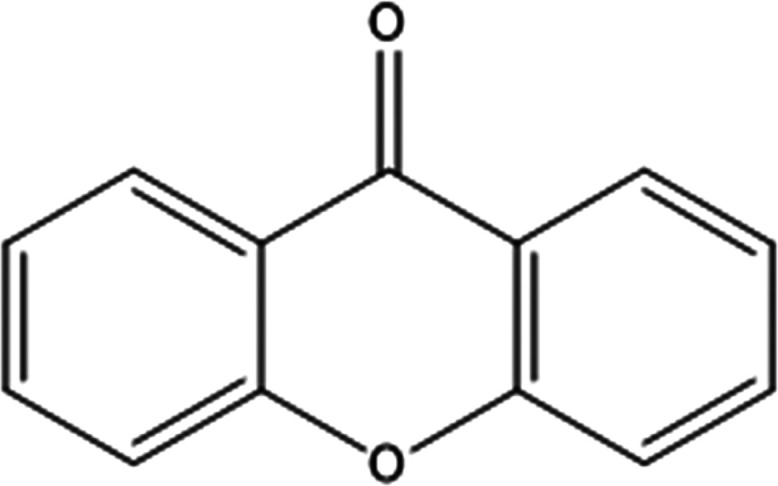
Molecular structure of xanthone (9H-xanthen-9-one).

The raw material used in this work was xanthone obtained from Alfa Aesar UK (99% purity; molecular structure shown in Figure 1). The recrystallization solvents (99% purity) were all purchased from Fisher Scientific and included acetone, methanol, ethanol, 2-propanol (IPA), toluene, ethyl acetate, heptane, and cyclohexane. Dimethyl sulfoxide (DMSO) and acetonitrile were purchased from Merck, UK (99% purity), whereas deionized water was obtained via a Milli-Q water purification system, Merck, UK.

### Solubility Measurement

Solubility studies were conducted using a thermogravimetric method^40^ and 10 common laboratory solvents: acetone, methanol, ethanol, 2-propanol (IPA), toluene, ethyl acetate, heptane, cyclohexane, acetonitrile, and deionized water. The solvents ranged in polarity from nonpolar (cyclohexane) to polar (water).

Slurries were prepared by adding excess xanthone to 10 mL of the solvent used. The vials were then placed in a temperature-controlled oven set at 20 °C for a minimum of 48 h to allow the sample to reach equilibrium. A syringe was used to take a 2 mL aliquot of solution. The extracted solution was filtered through a 0.45 μm filter and weighed in a Petri dish. The Petri dishes were left at room temperature for the solvent to evaporate and were weighed daily until a constant mass was achieved. The solubility of xanthone was measured in the solvent mixtures outlined in Table 1: acetone/water, IPA/acetone, and IPA/water solutions at different weight ratios. The solubility of the xanthone was also measured in acetone/water mixtures between 20 and 50 °C at 5 °C intervals using the thermogravimetric method mentioned above.

**Table 1:** Solvent Mixtures Used in Slurry Experiments to Determine Changes in the Solubility of Xanthone in Mixed-Solvent Systems

acetone/water (w/w)	IPA/acetone (w/w)	IPA/water (w/w)
0:100	0:100	0:100
10:90	10:90	10:90
20:80	20:80	20:80
30:70	30:70	30:70
40:60	40:60	40:60
50:50	50:50	50:50
60:40	60:40	60:40
70:30	70:30	70:30
80:20	80:20	80:20
90:10	90:10	90:10
100:0	100:0	100:0

### Recrystallization by Solvent Evaporation

Solid-form screening of xanthone was performed using solvent evaporation in 10 different solvents (water, ethanol, isopropanol, acetone, heptane, toluene, cyclohexane, methanol, ethyl acetate, and acetonitrile). Three levels of initial supersaturation were used. The xanthone was weighed into 5 mL vials and 2 mL of the appropriate solvent was added to each of the vials and stirred with an IKA RCT Basic magnetic stirrer to dissolve the solid.^41^ When the compounds had dissolved, vials were covered in parafilm with a series of small holes to allow slow evaporation of the solvent, as it was left at ambient temperature. The resulting crystals were filtered under a vacuum and dried at room temperature.

### Cooling Crystallization

Recrystallization was carried out in a 300 mL jacketed vessel equipped with an overhead stirrer and a PTFE impeller to mix the system. The impeller had four pitched blades and was placed approximately 10 mm above the bottom of the vessel to ensure adequate mixing of the crystallization medium. The temperature inside the vessel was controlled by a PT-100 temperature sensor that was connected to a Huber Ministat 230 thermostat (Huber, UK), and was measured every 5 s. An Optek C4000 turbidity probe (Optek-Danulat GmbH) was added to monitor changes in the light absorbance and transmittance throughout the crystallization experiment so that nucleation and growth could be monitored in real time. The probe tip was orientated toward the flow of the crystallization mixture to allow representative measurements to be made and to prevent fouling phenomena. A vertical condenser was added on top of the vessel to prevent the loss of solvent through evaporation. The stirrer was set at 200 rpm. This setting was selected because it was high enough to ensure that the xanthone particles were suspended throughout the experiment and low enough to minimize the formation of bubbles, as well as crystal breakage. An overhead stirrer was used instead of magnetic agitation to reduce the rate of crystal attrition.^42^ Saturated solutions of xanthone were prepared by dissolving it in a specific solvent mixture (e.g., acetone/water, DMSO/water, acetonitrile/water, IPA/water) at 45 °C. The solution was left stirring until complete dissolution of the solid occurred. Supersaturation was achieved by reducing the solution temperature, and nucleation was observed to occur when the solution turned cloudy and the transmittance values read by the turbidity probe started to drop significantly. The temperature was decreased from 45 to 5 °C at a rate of −1 °C/min using the Huber thermoregulator. Samples were collected at the end of the crystallization experiment for analysis by optical microscopy to observe the size and morphology of the crystals. The remaining crystals were recovered by vacuum filtration using Whatman filter paper (no.1) and air-dried at room temperature for a minimum of 24 h before analysis by scanning electron microscopy (SEM), differential scanning calorimetry (DSC) and powder X-ray diffraction (PXRD). Table 2 summarizes the ratios of acetone and water used for this set of crystallization experiments together with the cooling rates and the loading of xanthone used for each experiment.

**Table 2:** Summary of the Acetone/Water Ratios and Cooling Rates Used to Prepare Xanthone Crystals via Cooling Crystallization at the 300 mL Scale

solvent ratio w/w (acetone/water)	cooling rate (°C/min)	xanthone loading (g/100g of solvent)
100:0	–1	1.60
100:0	–0.5	1.60
100:0	–0.25	1.60
100:0	–0.125	1.60
90:10	–1	1.40
90:10	–0.5	1.40
90:10	–0.25	1.40
80:20	–1	1.05
80:20	–0.5	1.05
80:20	–0.25	1.05
70:30	–1	0.71
60:40	–1	0.37
50:50	–1	0.28
50:50	–0.5	0.28
50:50	–0.25	0.28
40:60	–1	0.20

### Antisolvent Crystallization

Saturated solutions of xanthone in acetone were prepared in a jacketed crystallization vessel at 45 °C. Each solution was kept at 45 °C long enough for the xanthone to dissolve. Then, supersaturation was achieved by adding water to the solution as antisolvent. An Anton Paar syringe pump was used to add water to the solution at a controlled rate. The final solvent ratio for acetone to water mixtures was kept constant at a weight ratio of 60:40 and the antisolvent was added at the rates of 1.88, 3.75, and 7.50 mL/min, respectively. Crystallization was allowed to continue for a further hour after the addition of antisolvent was completed. The crystals produced were recovered by vacuum filtration and left to air-dry at room temperature for a minimum of 24 h.

### High-Speed Homogenization

A further set of experiments were conducted using an IKA T25 Ultra-Turrax high-speed homogenizer. The xanthone solutions were prepared by adding 6.4 g of xanthone to 400 mL of acetone (16 mg/mL) under magnetic stirring. Each solution was then transferred to a beaker, placed under the homogenization head, and agitated at 13,500 rpm. Water at room temperature was added to the stirring solution and homogenized for a further 3 min, without controlling the temperature. The samples were vacuum-filtered immediately after homogenization, and the crystals were air-dried at room temperature for a minimum of 24 h before analysis.

### Jet Homogenization

Saturated xanthone solutions were prepared in acetone at 16 mg/mL and transferred to one of the two chambers in the jet homogenization head.^43^ Water was placed in the second chamber. The amount of water used was varied to produce final solvent mixture weight ratios of 50:50 and 60:40 solvent to antisolvent ratio. The solutions were passed through the high-pressure Leeds jet homogenizer (University of Leeds, UK)^44^ for 1 pass at 300 bar. The samples were vacuum-filtered immediately after homogenization, and the crystals were air-dried at room temperature for a minimum of 24 h before analysis. All steps of the procedure were carried out at ambient temperature.

### Optical Microscopy Particle Characterization

The crystal size and shape of each sample produced in the aforementioned crystallization experiments were observed via a Bressler binocular microscope at X4, X10, and X40 magnifications, and the images were recorded with a Sony IMx378 Exmor RS camera (12 Mpixels and an F/20 aperture) (Sony Semiconductor Manufacturing Corp, Japan). The image data collected was analyzed using ImageJ version 1.52a.^45^ For each sample, the length and width of around 700 crystals were measured, and the mean aspect ratio was calculated.

### Scanning Electron Microscopy (SEM)

Dry crystal samples were imaged using a Benchtop scanning electron microscope (Hitachi TM3030Plus) at magnifications ranging from 100× to 6000×. Samples were arranged on Leit adhesive carbon tabs attached to SEM specimen stubs. Before measurement, the prepared SEM stubs were sprayed with antidust spray to remove excess material that was loosely attached. The images were analyzed using ImageJ version 1.52a.^45^ The length and width of around 700 crystals were measured and the mean crystal length and aspect ratio were calculated.

### Single-Crystal X-ray Diffraction

X-ray diffraction data from a single xanthone crystal were collected on an Agilent SuperNova diffractometer at 130 K. The diffractometer was equipped with an Atlas CCD detector, which in turn was connected to an Oxford CryoStream low-temperature device. The device used mirror monochromated Cu Kα radiation (λ 1.54184 Å) coming from a microfocused X-ray source. The xanthone single crystal had dimensions of 0.17 mm × 0.11 mm × 0.07 mm. Higher resolution data of xanthone single crystals were also collected at the X-ray diffraction beamline (XRD1) of the Elettra Sincrotrone Trieste (Italy). The crystals were dipped in NHV oil (Jena Bioscience, Jena, Germany) and mounted on a goniometer head with Kapton loops (MiTeGen, Ithaca). Complete data sets were collected at different temperatures from 130 to 300 K every 10 K in order to carefully determine the polymorphic landscape of xanthone. A temperature ramp of 6 K/min was used for each step. Data were acquired using a monochromatic wavelength of 0.700 Å on a Pilatus 2 M hybrid-pixel area detector (DECTRIS Ltd., Baden-Daettwil, Switzerland). The crystal structure was solved by intrinsic phasing using the SHELXT^46^ computer algorithm, which considers the space group symmetry and peak position based on single-crystal reflection data. Further refinement of the data was performed using a least-squares technique based on a full matrix (*F*^2^) using SHELXL2016.^47^ The measured patterns were compared with the simulated powder patterns for xanthone in the CCDC Mercury software v.3.10 visualization software.^48^ Additionally, a principal component analysis (PCA) was conducted on the synchrotron data to verify the presence of phase transition events in the range of temperatures analyzed. The extrapolation of PXRD diffractograms from SXRD data at the different sampling temperatures was performed by using a multicore bilinear interpolation with CrysalisPro 42.49.^49^ MATLAB 2021a was used for the preprocessing and the analysis (using the default *pca* function). Patterns were smoothed using the Savitzky-Golay filter (default MATLAB function *sgolayfit*) with third order and frame length of 7 points. The default MATLAB function *normalize* was then used to normalize data before the PCA.

### Powder X-ray Diffraction (PXRD)

Powder XRD was used to characterize the xanthone samples prepared using the crystallization methods listed above (solvent evaporation, cooling, and antisolvent crystallization). A Panalytical X’Pert PRO was set up in the Bragg-Bentano mode using Cu Kα radiation (λ = 1.54184 Å). The sample was scanned at a 2θ range between 4 and 55° (40 kV, 40 mA) with a step size of 0.026° and time per step of 57 s.

### Differential Scanning Calorimetry (DSC)

The thermal properties of xanthone were observed by using a Mettler TOLEDO DSC-1 calorimeter. The differential scanning calorimeter was calibrated using indium. The lid was crimped in place, and the samples were heated from 25 to 250 °C at a rate of 10 °C/min. The heat flow was measured in mW, and the position of the maximum of the melting peak was used to determine the melting point.

### Computational Modeling

All of the xanthone .cif files (ZZZTXI13, ZZZTXI14, and JP_Xanth_A60W40_twin1_hklf4) were opened in the CCDC Mercury software v.3.10^48^ and the structures were reviewed to check that they contained the hydrogen atoms in the correct positions and appropriate bond types. The packings of the xanthone molecules in both the monoclinic and orthorhombic unit cells were compared. The Bravais–Friedel–Donnay–Harker (BFDH) morphology was viewed using the CSD-Materials tab, and the option for Visual Habit was selected. Visual Habit^50^ uses the attachment energy model to calculate the predicted morphology of the examined crystal structure. The settings used for this calculation were a limiting radius of 30 Å, the Dreiding II force field, and the Evjen method to calculate the electrostatic interactions. The modified morphology was viewed in the main window in Mercury. The outputs of the calculation were the total lattice energy and a breakdown of the intermolecular interactions that contribute to the lattice energy. The main facets, their % surface area, and key intermolecular interactions were provided. The interactions were ranked, and the main synthonic interactions were identified.

## Results and Discussion

### Solubility

**Figure 2:**
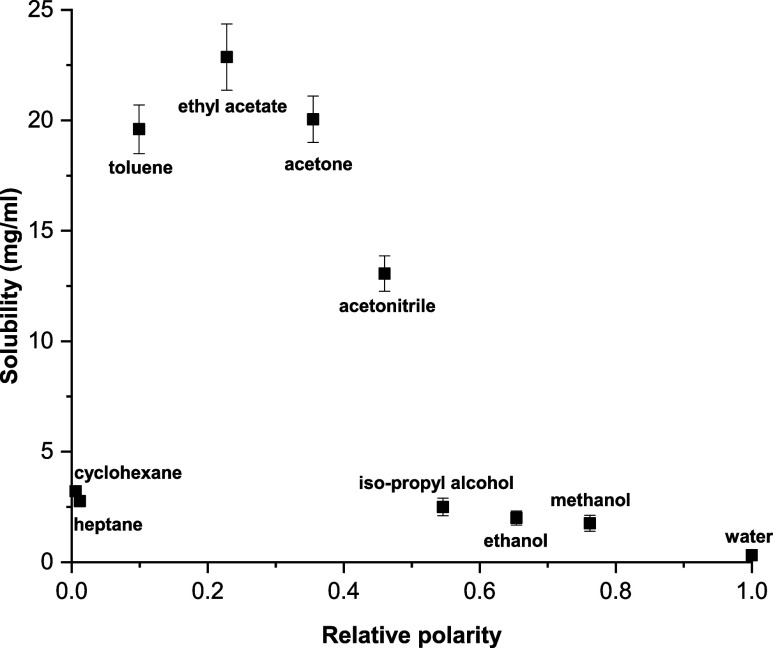
Summary of the solubility of xanthone at ambient temperature in common laboratory solvents.

Figure 2 displays the solubility of xanthone in a range of common laboratory solvents that differed in their polarities.^41^ The values were determined using a thermogravimetric method and revealed that xanthone had limited solubility (<4 mg/mL) in both highly nonpolar (e.g., cyclohexane) and highly polar solvents (e.g., methanol and water). The highest solubility (>12 mg/mL) was found to be in ethyl acetate, acetone, and toluene that have lower polarities (0.1–0.4) compared to water (1.0). Acetone was selected as the primary solvent for the cooling and antisolvent experiments since this was the most cost-effective of the solvents identified previously. The limited solubility of xanthone in water made this an ideal anti-solvent.

**Figure 3:**
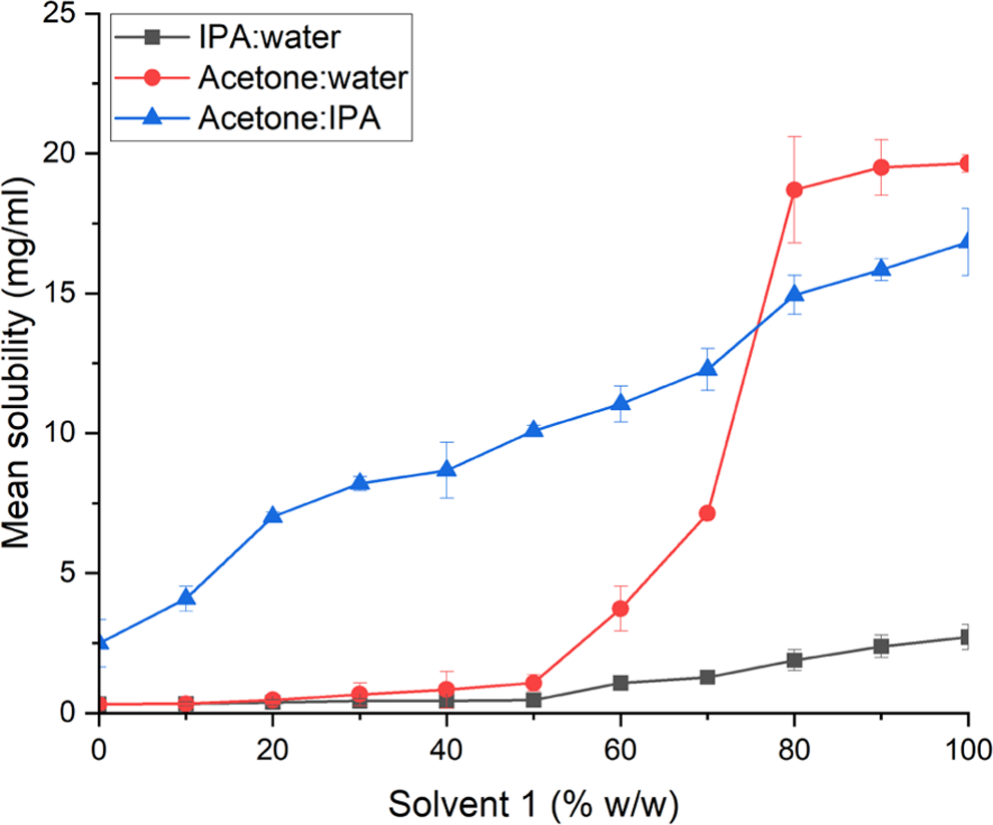
Solubility of xanthone at different solvent ratios (% w/w) with solvent 1 and solvent 2 for each pairing indicated in parentheses: (a) IPA (solvent 1)/water (solvent 2), (b) acetone (solvent 1)/IPA (solvent 2), and (c) acetone (solvent 1)/water (solvent 2).

Solubility measurements were also performed for solvent mixtures to aid the design of antisolvent crystallization experiments (Figure 3).

Xanthone presented a high degree of solubility (>18 mg/mL) when no water was added to the solvent. As the level of water increased the solubility began to reduce. The solubility drops to <8 and <2 mg/mL when 30 and 70% w/w water are used, respectively. Solubility studies of acetone:IPA mixtures indicate that IPA does not perform well as an antisolvent for this system. Xanthone remains more soluble in this system over the whole range of ratios investigated when compared to the same ratios of both acetone and IPA in water.  Based on these observations the acetone/water mixture was the recrystallization medium of choice because the solubility of xanthone is a strong function of the amount of acetone present. Hence, acetone/water mixtures produced the highest levels of supersaturation that can be achieved and the highest yields of crystals.

Solubility measurements as a function of temperature (Supporting Information, Figure S1) revealed that as the temperature was increased from 20 to 50 °C, the solubility of xanthone increased markedly in pure acetone. In mixtures of acetone and water, the temperature effect was less significant and inversely proportional to the amount of water in the solvent.

### Recrystallization by Solvent Evaporation

Xanthone crystals were successfully recrystallized from all of the solvents studied except for pure water, due to the very low solubility of xanthone in this solvent. Xanthone was also very soluble in dimethyl sulfoxide (DMSO), >25 mg/mL, but this solvent was unsuitable for evaporation experiments due to its high boiling point. DMSO was therefore only used in cooling and antisolvent recrystallizations.

**Figure 4:**
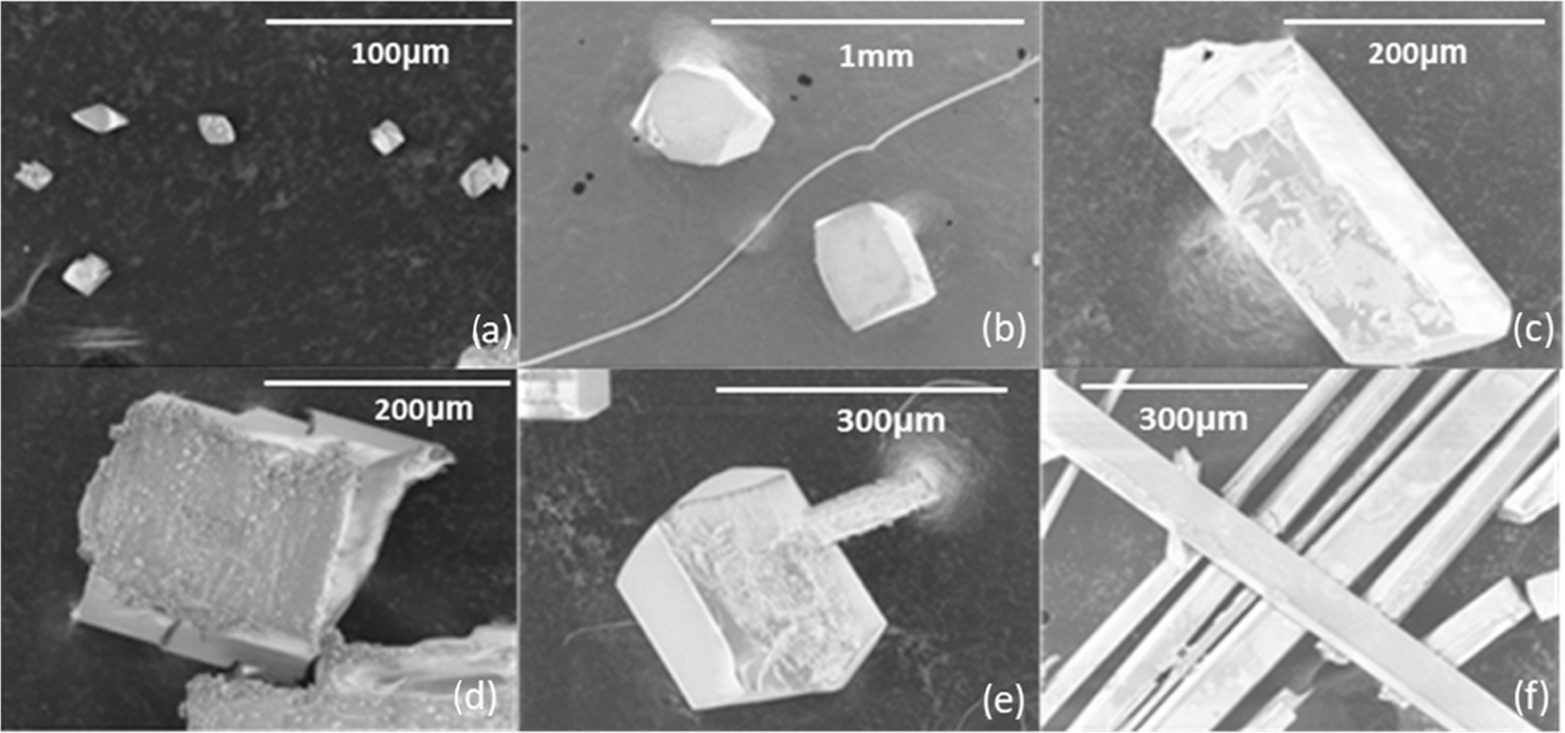
SEM images of xanthone crystals recrystallized from (a) heptane, (b) acetonitrile, (c) ethyl acetate, (d) methanol, (e) ethanol, and (f) toluene.

Figure 4 displays the morphology of xanthone crystals recrystallized from solvents with different polarities. The crystals recovered from toluene and acetone had a needle-like morphology, whereas crystals extracted from heptane and acetonitrile had a more prismatic shape.

**Figure 5:**
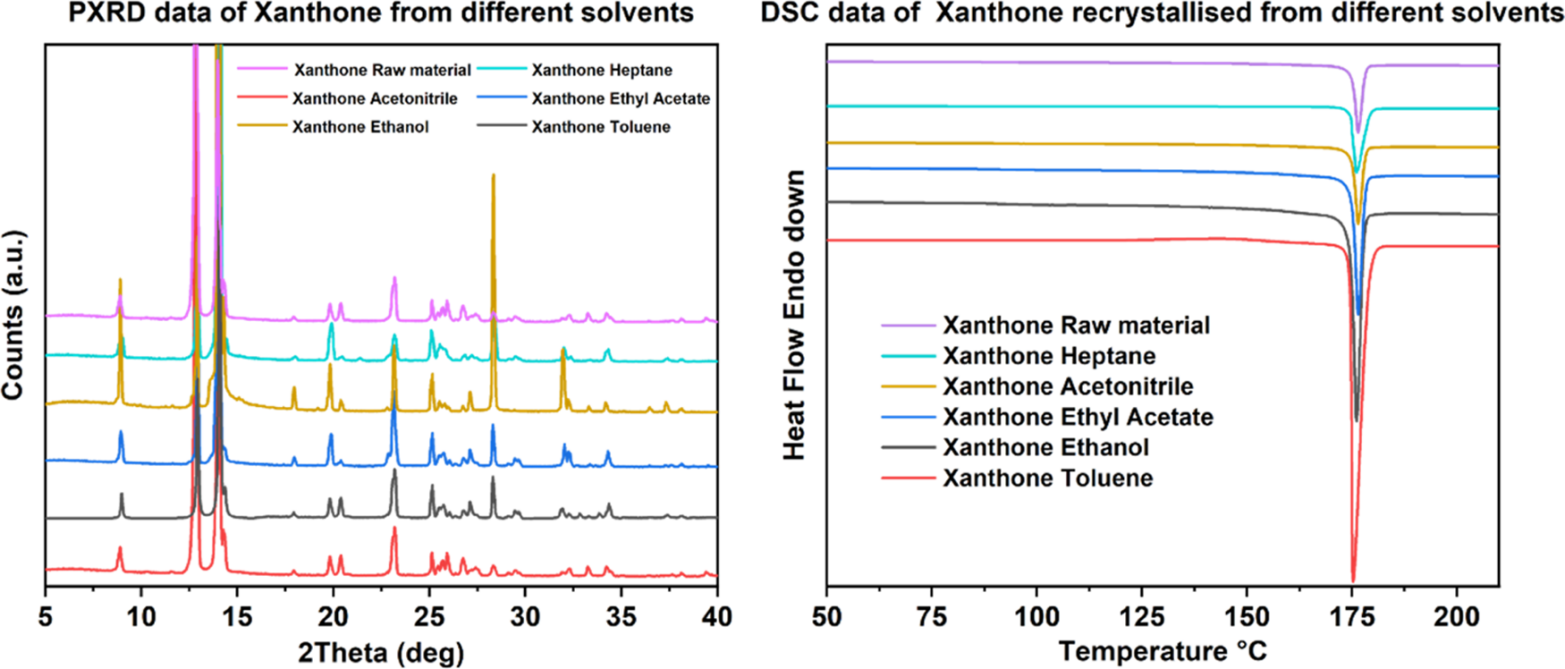
PXRD and DSC profiles from xanthone recrystallized from a range of solvents using the solvent evaporation technique.

Despite differences in crystal shape, both powder XRD and DSC analyses (Figure 5) revealed that all of the crystals were of the same solid form, irrespective of the solvent type from which they were recrystallized. This observation supports the work of Saršu̅ns et al.,^35^ where only one solid form of xanthone was crystallized at ambient temperature, via evaporation.

**Figure 6:**
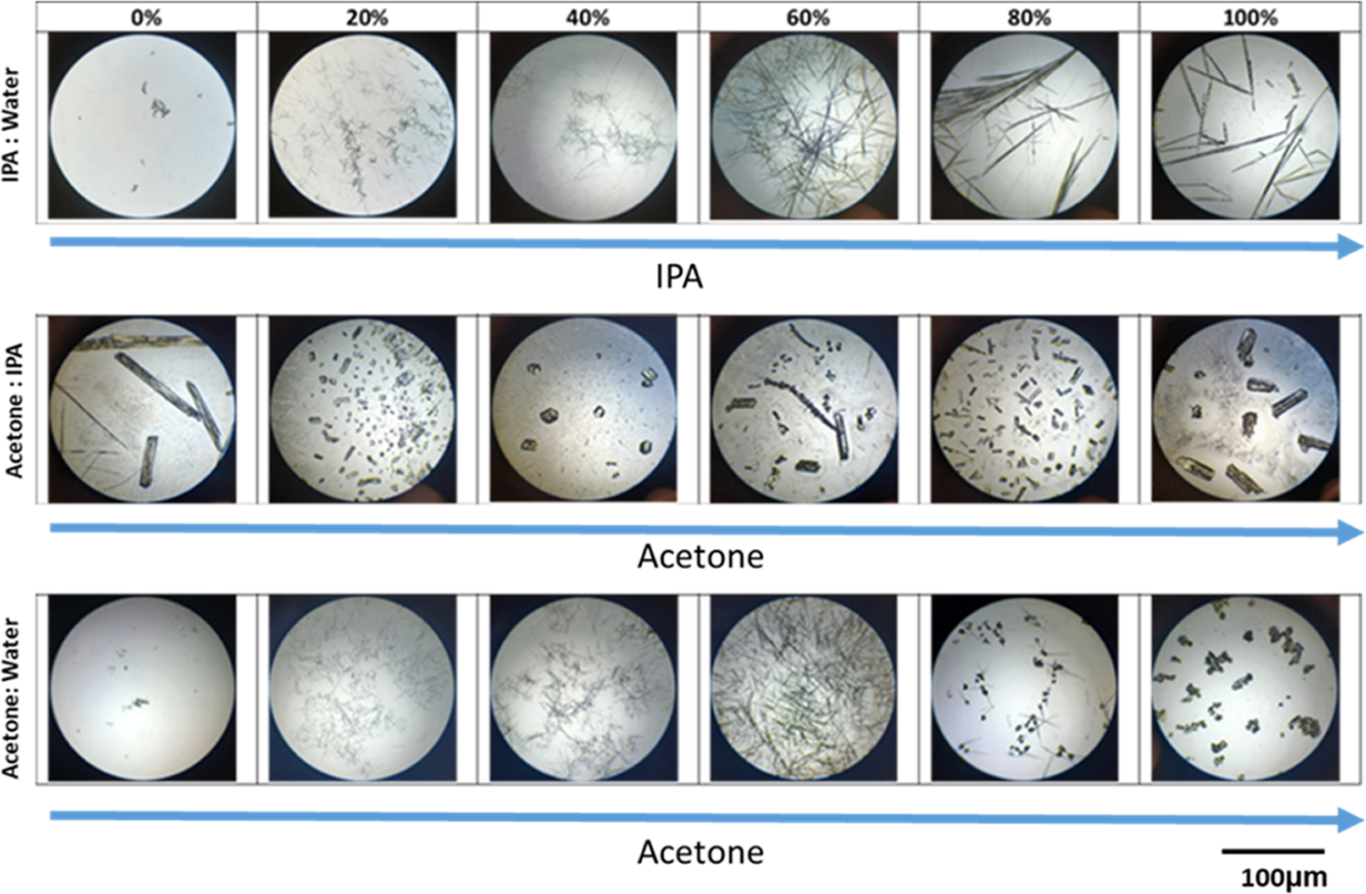
Optical microscopy of the Petri dishes containing the crystals recrystallized after the thermogravimetric analysis of xanthone in different solvent ratios of IPA/water, acetone/IPA, and acetone/water.

Figure 6 shows the effect of different solvent mixtures on the crystal morphology of xanthone. The crystal habit in the IPA–water system was the least affected by the amount of water in the solvent. The crystals had a needle-like morphology, and differences in size were due to the different levels of supersaturation that could be reached with changing solvent composition; in fact, higher IPA concentrations allowed more xanthone to be dissolved before evaporation (e.g., higher solubility in IPA than water).

Xanthone crystals recovered from the acetone/IPA and the acetone/water systems presented a broader shape and size distributions with rods and needles coexisting together and the presence of considerable secondary nucleation. This is probably due to the very different volatility of the two solvents that resulted in different solvent compositions during the evaporation, and hence different solubilities and supersaturation trends over time. Such irregular supersaturation generation affected the kinetics of secondary nucleation and facet-specific growth, generating different shapes.

### Cooling Crystallization

**Figure 7:**
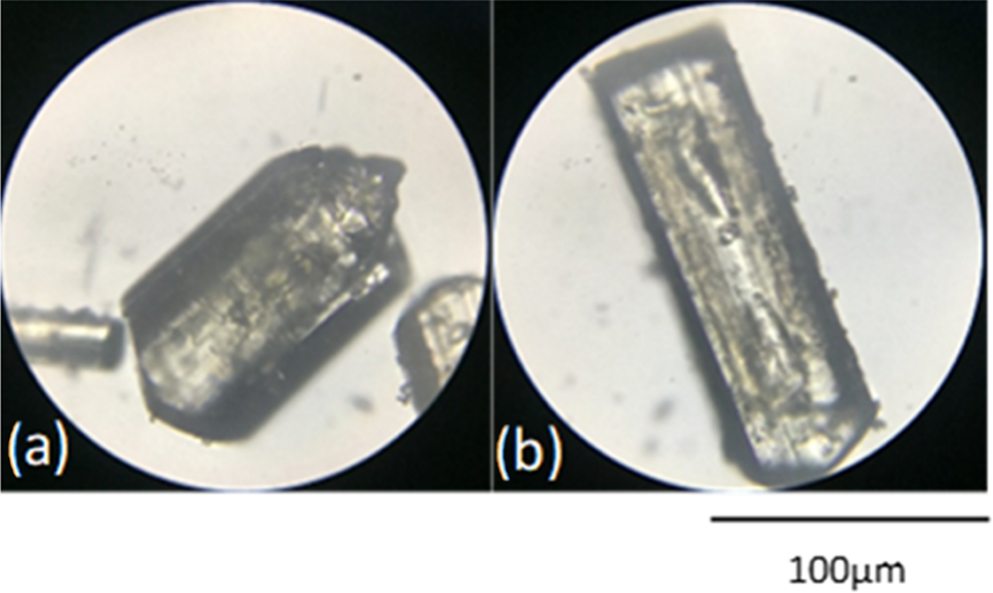
Effect of rate of filtration on final crystal size (crystals recovered from 100% acetone). Fast (a) versus slow (b) filtration rate.

Cooling crystallization was used to successfully produce xanthone rods of >200 μm in size due to the controlled generation of supersaturation via slow temperature decrease. Even for this set of experiments, only one polymorphic form was produced, based on PXRD and DSC analysis. The process was repeatable and routinely produced crystals of the same size and shape distributions and solid form. Increasing the rate of cooling from −0.25 °C to −1 °C/min had a limited effect on the final size distribution while the speed of filtration after the end of the experiments performed in acetone was critical in preventing further crystal growth via evaporation, as shown in Figure 7.

**Figure 8:**
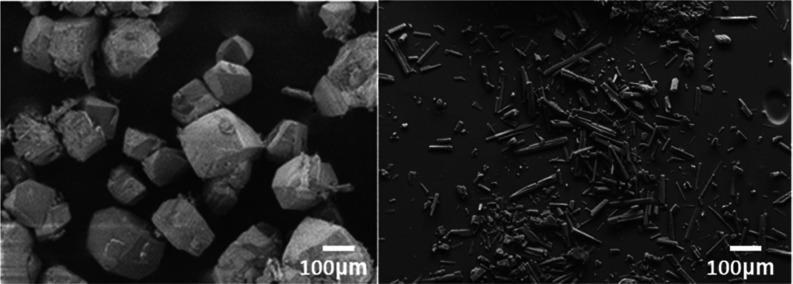
Comparison of the prismatic crystal morphology from acetonitrile/water cooling crystallization (left) compared to needle-like morphology recovered from acetone:water solutions (right).

The effect of solvent composition on the crystals produced via cooling crystallization was also investigated. Figure 8 shows the changes in crystal morphology during cooling crystallization when a mixed-solvent system was used. DMSO/water and acetonitrile/water solvent mixtures produced crystals that were prismatic, whereas crystals created from acetone/water mixtures were needle-like. Different, facet-specific solute–solvent interactions among these solvents might be responsible for this behavior.

### Antisolvent Crystallization

Initial crystallization experiments involved the addition of pure water at different ratios to a xanthone solution in a 300 mL reaction vessel, stirring at 200 rpm. Every crystallization experiment resulting in primary nucleation was successful and began when 75% of the total water had been added. The resulting crystals were around 100 to 500 μm in size (i.e., length) and adopted either a prismatic or needle morphology depending on the ratio of water to acetone. PXRD and DSC confirmed the same solid form, despite the different morphologies achieved. It is worth noting that water in the final solvent mixture resulted in smaller and longer needles compared to higher percentages of acetone. Additionally, the shape and size distribution were more homogeneous compared to those obtained via evaporation (Figure 6); this is due to the rapid de-supersaturation, which is typical of crystallization processes with rapid antisolvent addition.

### High-Speed Homogenization

**Figure 9:**
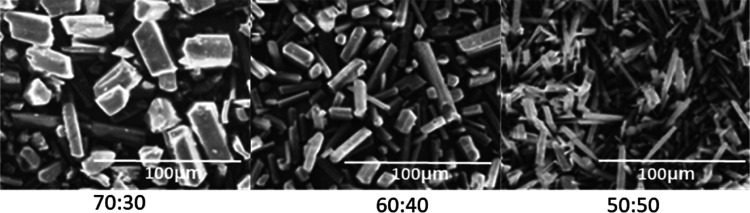
Comparison of the aspect ratio of crystals recrystallized from acetone:water mixtures at varying weight ratios (70:30, 60:40, and 50:50) using antisolvent crystallization and a high-speed Ultra-Turrax homogenizer.

In order to further increase the supersaturation at the nucleation point and induce the formation of metastable crystal structures, an Ultra-Turrax homogenizer was used to increase the mixing speed for the antisolvent crystallization experiments. Initial experiments using acetone:water mixtures at ratios of 70:30, 60:40, and 50:50 w/w demonstrated that the amount of water added had a significant effect on the aspect ratio and size of the crystals obtained (Figure 9), but not on the solid form generated (same PXRD and DSC). The crystals prepared using acetone:water at a ratio of 70:30 were broader in size and shape distribution and presented a more prismatic habit, whereas crystals prepared at a ratio of 50:50 had a higher aspect ratio and became more needle-like. The average aspect ratios estimated from SEM images were around 1.8 (70:30) and 4.4 (50:50), respectively.

Even higher water concentrations than those of Figure 9 were used (60–90% w/w), and the same trend was observed, despite the considerably lower solubility of xanthone in such a high amount of water in the solvent mixtures. Each of the solvent ratios investigated produced crystals with a needle-like morphology and at 80–90% w/w water the needles became markedly thinner and shorter, the average length ranging from 5 to 30 μm. If the crystals were not filtered immediately after preparation or the sample was filtered too slowly they continued to grow via evaporation (the average length increased even up to 50–100 μm).

### Jet Homogenization

The smallest crystals obtained in this work were produced via jet homogenization (around 1.3–20 μm of average length), possibly because this technique allowed the fastest mixing between solvent and antisolvent, with the highest nucleation rate as high supersaturation values could be reached. The xanthone particles produced using this technique showed more variability in aspect ratio than those produced using cooling crystallization or with the Ultra-Turrax. Samples were prepared using acetone:water ratios of 80:20 and 20:80 w/w, the resulting xanthone crystals again contained crystals of two different morphologies, prisms and needles (Table 3), but the same crystal structure.

**Table 3:** Summary of the Morphological Composition (Prisms and Needles), Mean Length (and Standard Deviation, SD), and Aspect Ratio (AR) of Xanthone Samples Prepared Using 20% and 80% Water

			prisms (size and aspect ratio (AR))		needles (size and aspect ratio (AR))	
water (%)	prisms (%)	needles (%)	mean length (μm), mean AR		mean length (μm), mean AR	
20	36	64	1.28 (SD ± 0.86)	1.18 (SD ± 0.22)	3.04 (SD ± 2.61)	2.93 (SD ± 2.30)
80	16	84	1.74 (SD ± 1.71)	1.25 (SD ± 0.22)	4.56 (SD ± 3.30)	3.39 (SD ± 1.64)

As shown previously for the other antisolvent experiments, as the percentage of water was increased in the recrystallization medium the quantity of xanthone crystals with a prismatic morphology decreased and the quantity of crystals with a needle morphology increased. This led to increases in the mean aspect ratios of both the prisms (from 1.18 to 1.25 (±0.22)) and the needles (from 2.93 to 3.39 (± 1.97)) being observed. The mean crystal sizes (ie. maximum lengths) of the two morphologies were also different. The prismatic crystals were shorter (1.28–1.74 μm) when compared with the needles (3.04–4.56 μm).

### Polymorphism Investigation  Using Single-Crystal XRD

**Figure 10:**
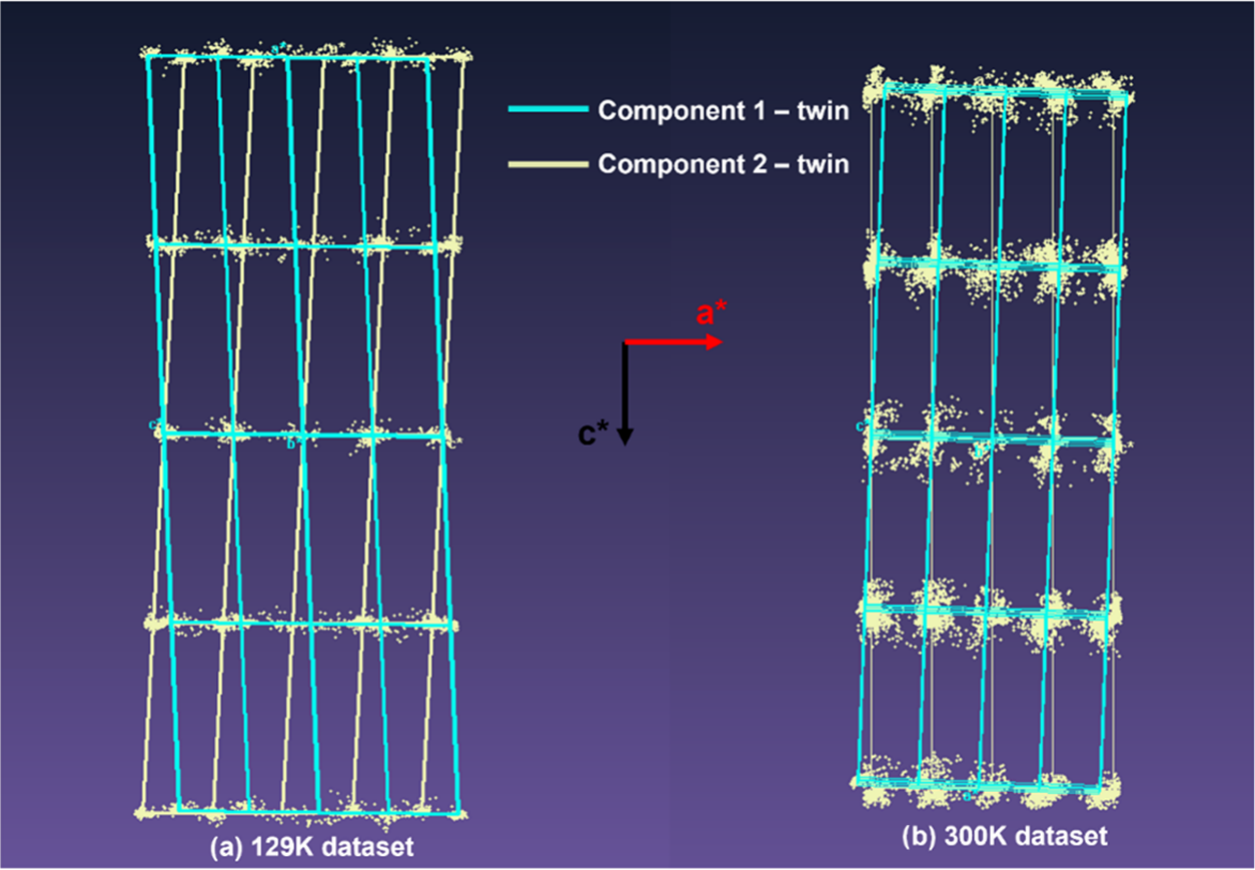
Ewald patterns of xanthone observed at 129 and 300 K.

Suitable single crystals of xanthone for single-crystal X-ray crystallography were obtained from antisolvent crystallization from a mixture of xanthone in 60% acetone and 40% water (% w/w). The crystal structure resolution at 300 K highlights as the best solution the orthorhombic *P*2_1_2_1_2_1_ space group. On cooling the same crystal to 130 K, the crystal structure resolution was different and could be described in the monoclinic space group *P*21 with a twinning defect. (Table S2 with crystallographic data is reported in the Supporting Information). However, by comparing the twinned monoclinic structure with the orthorhombic one reported by Onuma et al.,^30^ it is possible to highlight how the two structures share the same intermolecular interactions, the same crystal packing, and finally the same length of crystallographic axes (Figure S3, Table S3 in the Supporting Information). This is usually termed polysynthetic twinning, which allows the crystal to preserve its isometric shape by equalizing the displacement in every direction. This results in a *pseudopolymorphic* crystal that displays the appearance of isometric symmetry.^49,50^ This phenomenon was confirmed by the temperature ramp single-crystal X-ray diffraction measurements performed at the Elettra synchrotron (Table S3). Diffraction data were obtained every 10 K in the 300–129 K temperature range with a significantly appreciable increase in the β angle as the data collection is carried out at lower and lower temperatures (Table S3). This phenomenon is linked to a growing division of the diffraction spots and consequently to a better indexing of the diffraction patterns with the twinning law (Figure S2 in the Supporting Information). Here, we report in Figure 10 two Ewald patterns collapsed to the lattice range of two data sets measured at 129 and 300 K. For both data sets, it was possible to index the reflection spots with two geminated cells (blue and yellow traces in Figure 10) with the following transformation matrix [100 100 001]. For the data acquired at 129 K, the indexing of diffraction spots with the two geminated cells was unique. On the contrary, as the temperature increased, it was possible to notice how the size of the crystallographic axes remained almost unchanged, while the 2θ angle was gradually reduced from 92° to almost 90° (Table S3 in the Supporting Information). For the data set at a temperature of 300 K, in which the 2θ angle is close to 90°, it is possible to index the diffraction spots with an orthorhombic cell, but with a worse figure of merit (FOM) than the monoclinic. Moreover, by solving the crystal structure via adopting the two different crystal systems, it is possible to find that the discrepancy indices are very close to each other (8.6% and 9.4%, orthorhombic and monoclinic R factor, respectively). Analysis of the data from the single crystals, combined with analyses obtained by DSC and PXRD analysis, showed the presence of a single-crystal structure for xanthone at a wide range of temperatures.

The data for the *P*2_1_2_1_2_1_ space group can also be analyzed at a lower symmetry to give the *P*2_1_ space group, which is consistent with the single-crystal data in Table S1 in the Supporting Information.

The crystals are therefore monoclinic, with some weak reflections in the data leading to misidentification of the orthorhombic lattice.

**Figure 11:**
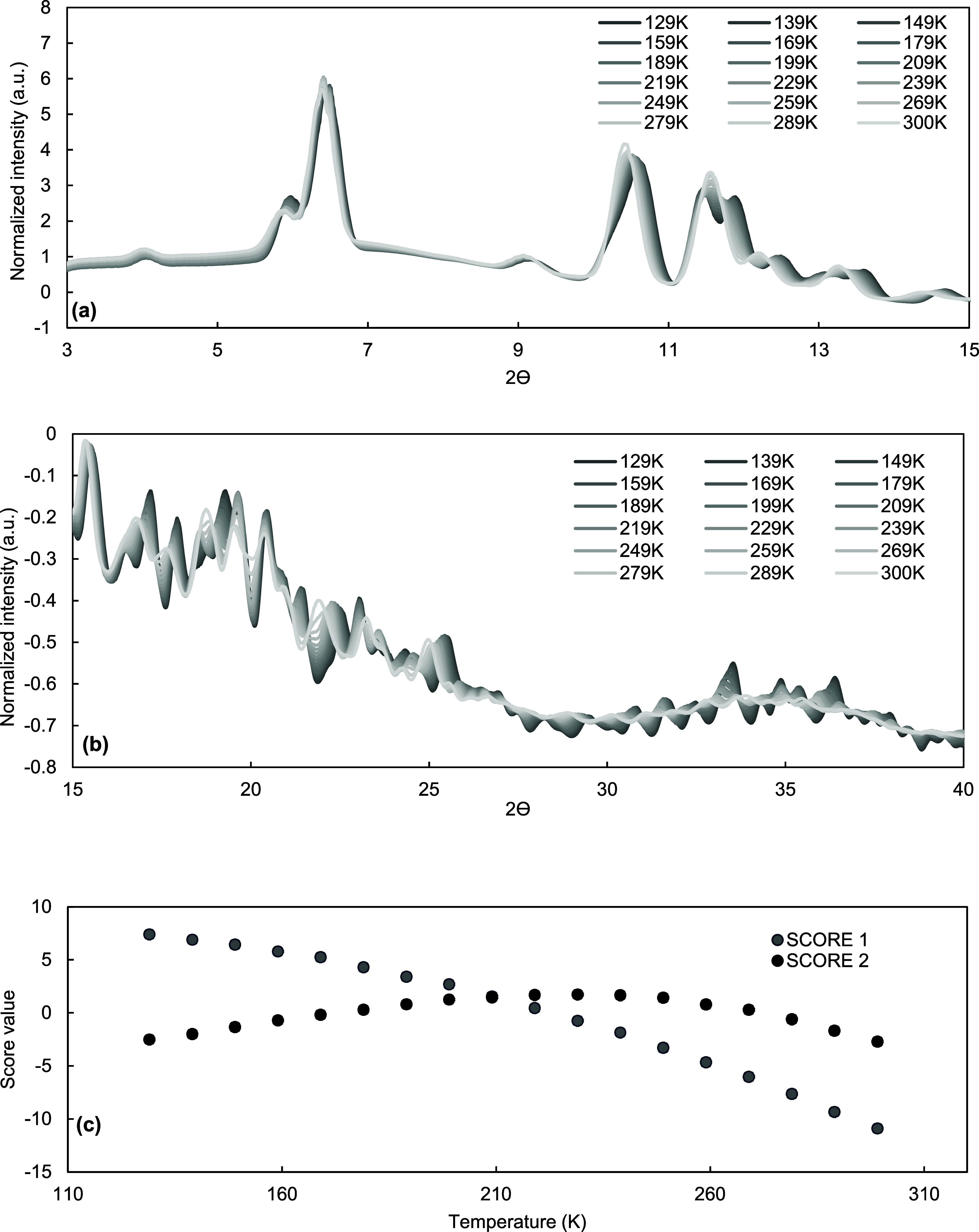
(a) Simulated smoothed and normalized PXRD patterns from single-crystal synchrotron XRD between 3 and 15° and (b) 15–40° 2θ, and (c) values of score 1 and score 2 of the PCA carried out on the data shown in (a) and (b).

In order to further verify the absence of transition events in the range of temperature studied, a PCA of the calculated PXRD patterns was performed on the single-crystal synchrotron XRD data. Figure 11a,b shows the PXRD patterns at different temperatures, where small differences are evident.

Figure 11c instead shows the scores of principal components 1 and 2 at different temperatures. These two components explain 99% of the variance of the whole data set, with score 1 explaining over 92% of the variance. Both scores do not show abrupt changes in trends as the temperature decreases, indicating the absence of a transition temperature that would indicate an enantiotropic system and, hence, the existence of two different polymorphs. Instead, a nonlinear trend for the PXRD data is evident, and probably related to the effect of temperature, which can cause vibration of the atoms around their crystallographic position. At room temperature it is possible that the twinning effect is difficult to understand because the crystal tends to maintain an isometric symmetry by mediating the displacement in every direction. This can lead to an interpretation of pseudo-polymorphic crystals that have an isometric symmetry.

### Computational Modeling

Analysis of the .cif files for the already existing ZZZTXI13 (orthorhombic), ZZZTXI14 (monoclinic), and the structure found in this work (JP_Xanth_A60W40_twin1_hklf4) revealed that all of the unit cells have similar packing arrangements, similar cell dimensions (Table S3 in the Supporting Information) and lattice energies of −103.9 to −104 kJ/mol, respectively. These similarities are further confirmation that xanthone is monomorphic rather than polymorphic, as previously stated.

Further evidence for xanthone being monomorphic but having different crystal habits from different solvents is also indicated by the lack of quantitative data in the literature on significant differences between the two polymorphs (e.g., melting point, solubility, and biological activity).

### CCDC Visual Habit

Xanthone (9H-xanthen-9-one) consists of a heterocyclic fused-ring system that is planar in nature. The planar conformation is stabilized by an extensive delocalized π-system.^38,39^ The two benzene rings are joined together by a central ring containing an ether group and a ketone group (Figure 1).^37,39^ The oxygen atoms from the ketone and ether groups make hydrogen bond acceptors, but there is a lack of good hydrogen bond donors on the molecule. These are principal factors to consider when studying the crystal structure and the intermolecular interactions that may occur at the surface of different facets of monoclinic xanthone.

**Figure 12:**
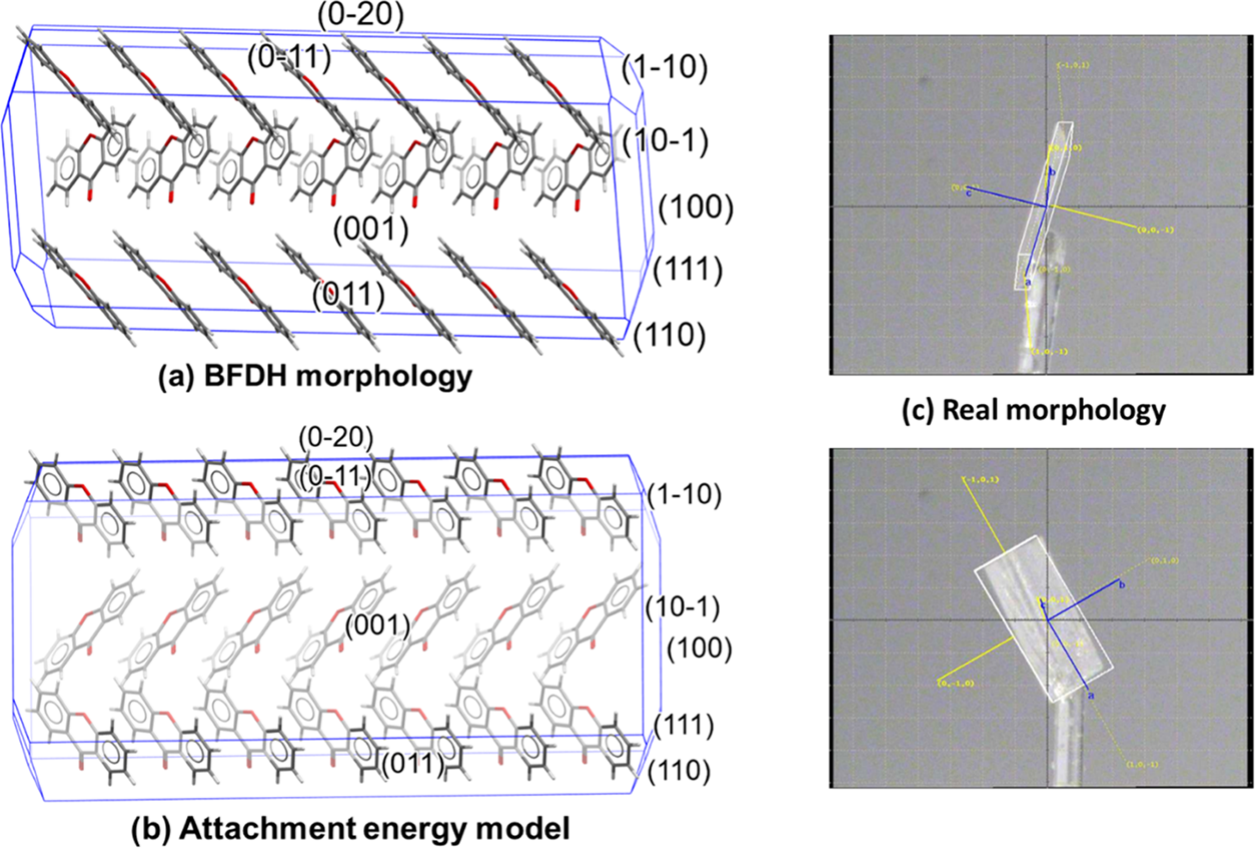
Predictions of the monoclinic crystal structure for xanthone crystals using (a) BFDH morphology calculations and (b) applying the attachment energy model using Mercury visualization software with the Visual Habit add-on (Edgington et al., 2006; Pickering et al., 2017).

**Figure 13:**
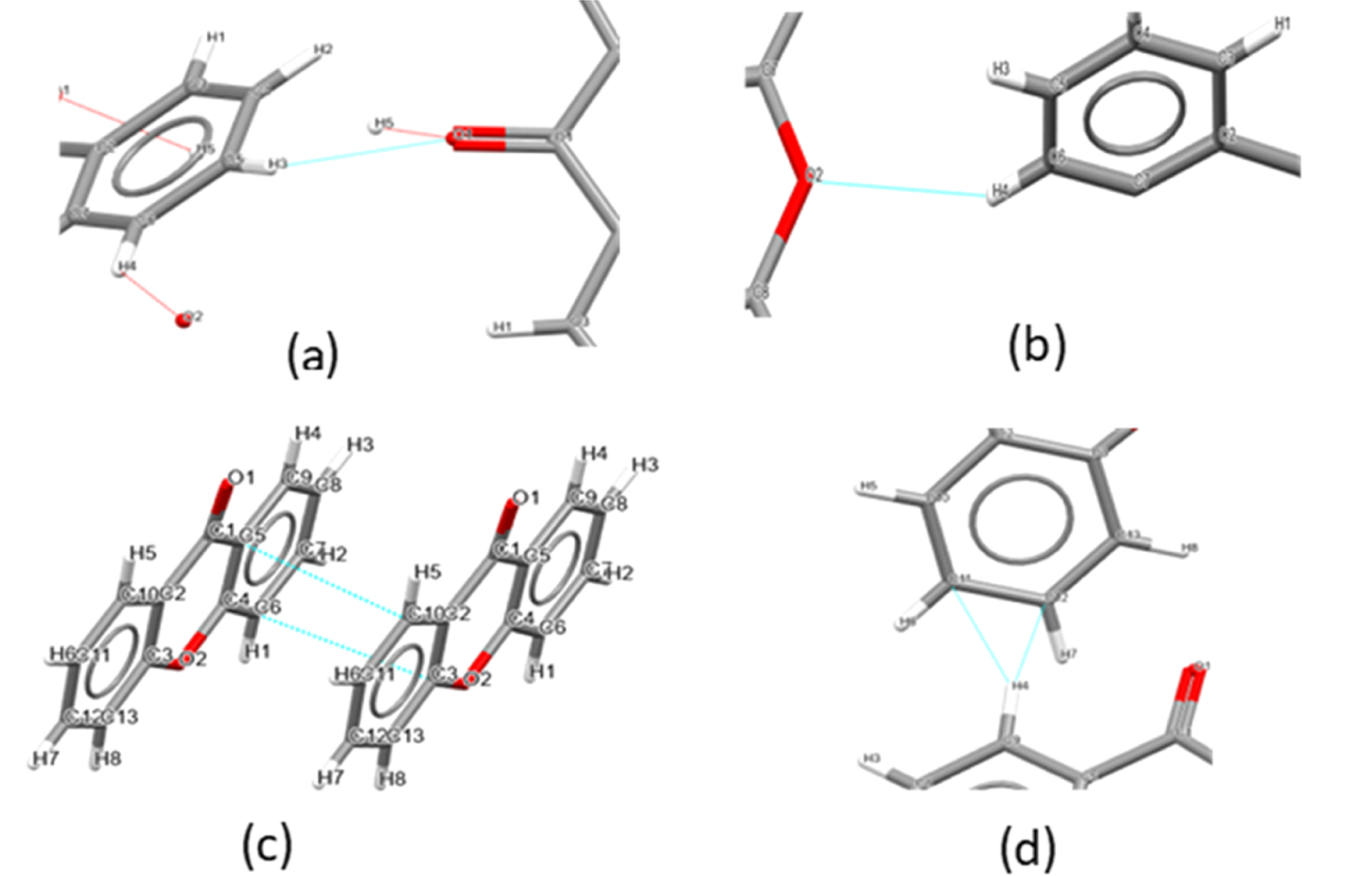
Summary of the key synthonic intermolecular interactions of xanthone: (a) O on the carbonyl group → H on the aryl group, (b) O from the biaryl ether group → H on the aryl group, (c) π/π stacking interactions between the C atoms of aryl groups of adjacent molecules, and (d) van der Waals attraction between aryl C atoms on one atom and the H atom on the adjacent molecule.

The Bravais–Friedel–Donnay–Harker (BFDH) morphology^48^ was used to predict the morphology of xanthone crystals. As shown in Figure 12, the predicted shape is needle-like, which is in agreement with the experimental results shown earlier. The BFDH model is the simplest one used to predict the morphology of a crystal from XRD data. It assumes that the most morphologically important facets have the greatest interplanar spacings. It considers the structure as it would be in a vacuum and does not consider the surrounding environment. It does not explain how the xanthone crystals will interact with specific solvents or more specifically which facets will form stronger intermolecular interactions with polar solvents and which will interact more with nonpolar solvents. More details about the nature and directionality of the intermolecular interactions characterizing each facet of xanthone crystals can be obtained using the attachment energy model in the Mercury tool, Visual Habit (Figure 13).^50^ The attachment energy model takes into consideration the main intermolecular interactions to build a more accurate prediction of the crystal morphology.

**Figure 14:**
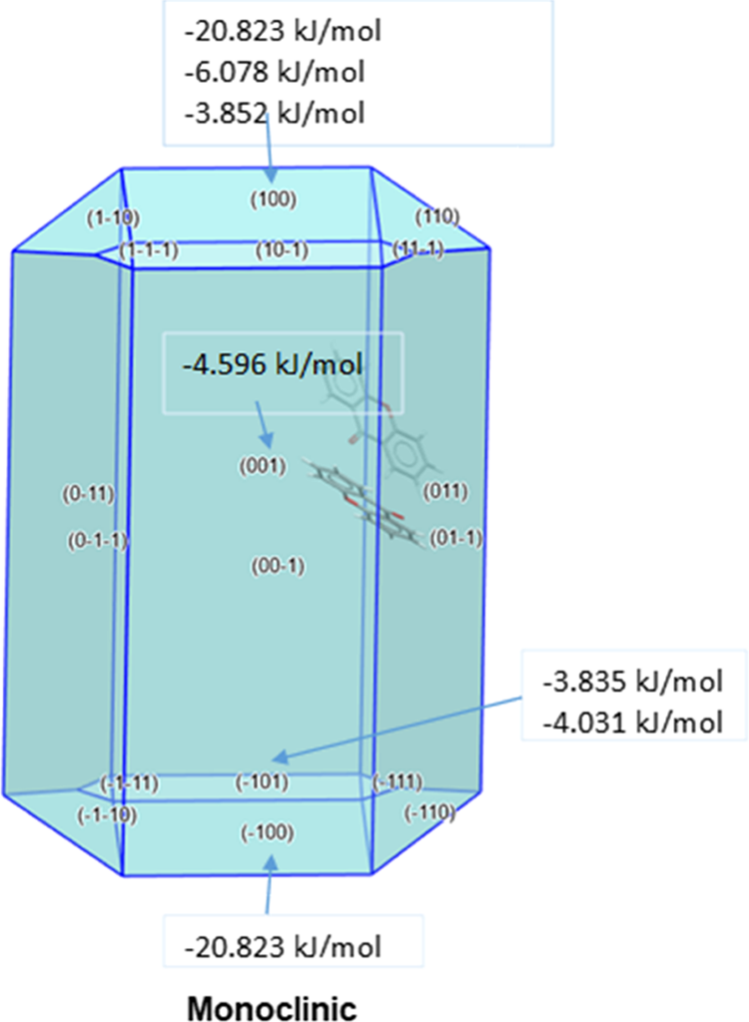
Summary of the contributions of different facets to the total lattice energy.

Figure 14 displays the main intermolecular interactions of xanthone. The first point of note is that xanthone does not form hydrogen bonds and therefore all of the interactions are short nonbonded contacts. Van der Waals forces contribute 98.8% to the total lattice energy, >80% of these interactions are due to π–π stacking interactions between the C atoms of aryl groups of adjacent molecules and the rest are due to van der Waals attraction between the aryl C atoms on one atom and the H atom on the adjacent molecule. The remaining interactions of the carbonyl or ether groups contribute little to the total lattice energy (1.2%).  They are due to weak electrostatic interactions between the O on the carbonyl group and the H on the aryl group and the O from the biaryl ether group → H on the aryl group.

The facets that grow the fastest in the xanthone crystals are the capping facets {100}, {110}, and {111}. They have the highest attachment energy, principally due to the van der Waals forces, but are less morphologically important, each facet contributing <5% of the total percentage area of the crystal (Figure 14). The lateral facets {001} and {011} by contrast, grow at a much slower rate and have lower attachment energies. The latter facets are expected to interact the most with solvent molecules and be the most surface active. They have the highest density of functional groups at the surface.

## Conclusions

In this paper, the polymorphic landscape of xanthone was investigated and clarified. Different solvents and crystallization techniques were used in an attempt to produce metastable structures. While PXRD patterns indicated the presence of a single solid phase for all experiments, conflicting results were observed with single-crystal XRD analysis carried out at ambient and low (130 K) temperatures. Ambient temperature measurements indicated the presence of an orthorhombic structure, while a monoclinic unit cell was better fitting the data collected at 130 K. High-resolution synchrotron data collected at different temperatures and analyzed with CrysalisPro 42.49 software, Olex2 1.5, and chemometric techniques (PCA) showed the presence of only one form, the monoclinic space group, *P*2_1_/*c* at every temperature. The assignment of the orthorhombic space group *P*2_1_2_1_2_1_ at the structures collected at higher temperatures was due to the *pseudosymmetry* effect that is due to mis-indexing of the diffraction spots with twinning unit cells. The evidence that we have presented indicates that 9H-xanthen-9-one (xanthone) is not polymorphic. Over the range of screening conditions evaluated, xanthone only forms one type of crystal lattice (i.e., monoclinic) but may exhibit different crystal habits dependent upon the preferential growth of facets in specific solvents and solvent mixtures.

## Supporting Information Available

Xanthone solubility in single- and mixed-solvent systems, crystallographic data including single-crystal structure refinement, diffraction images, crystal packing, and cell length and angle indexation (PDF)
