# Supplementary material for: Elucidating the Polymorphism of Xanthone: A Crystallization and Characterization Study
Source: Cryst Growth Des. 2024 Mar 28;24(8):3256–68. doi: 10.1021/acs.cgd.3c01506 (PMC11036362; doi:10.1021/acs.cgd.3c01506)
Supplement: Supplementary file 1 — cg3c01506_si_001.pdf [file cg3c01506_si_001.pdf]

Supplementary Information:

Elucidating the polymorphism of xanthone: A crystallization and characterization study

*Janine Andrea Preston[1], Emmanuele Parisi[2], Brent Murray[3], Arwen I.I Tyler[3], Elena Simone[2,3]**

[1] School of Chemical and Process Engineering, University of Leeds, Leeds LS2 9JT, United Kingdom

[2] Department of Applied Science and Technology (DISAT), Politecnico di Torino, 10129 Torino, Italy

[3] Food Colloids and Bioprocessing Group, School of Food Science and Nutrition, University of Leeds, Leeds LS2 9JT, United Kingdom

*Corresponding: elena.simone@polito.it

SI. No.	Title	Page No.
**1**	**Figure S1** Solubility of xanthone at different temperatures in single solvent and mixed solvent systems	S2
**2**	**Table S1** Single crystal data and structure refinement for xanthone	S3
**3**	**Figure S2** Diffraction images of xanthone crystal at a) 129 K and b) at 300K.	S4
**4**	**Figure S3** Crystal packing of xanthone. A) view along the *b* axis b) View along the *a* axis. Weak hydrogen bond interactions are represented in blue dashed lines.	S4
**5**	**Table S2** Crystallographic data comparison between orthorhombic and monoclinic xanthone. The data are taken from the Crystallographic Structure Database (CSD).	S4
**6**	**Table S3** Cell length and angle indexation of the SXRD xanthone dataset. Datasets are sorted by increasing temperature.	S5

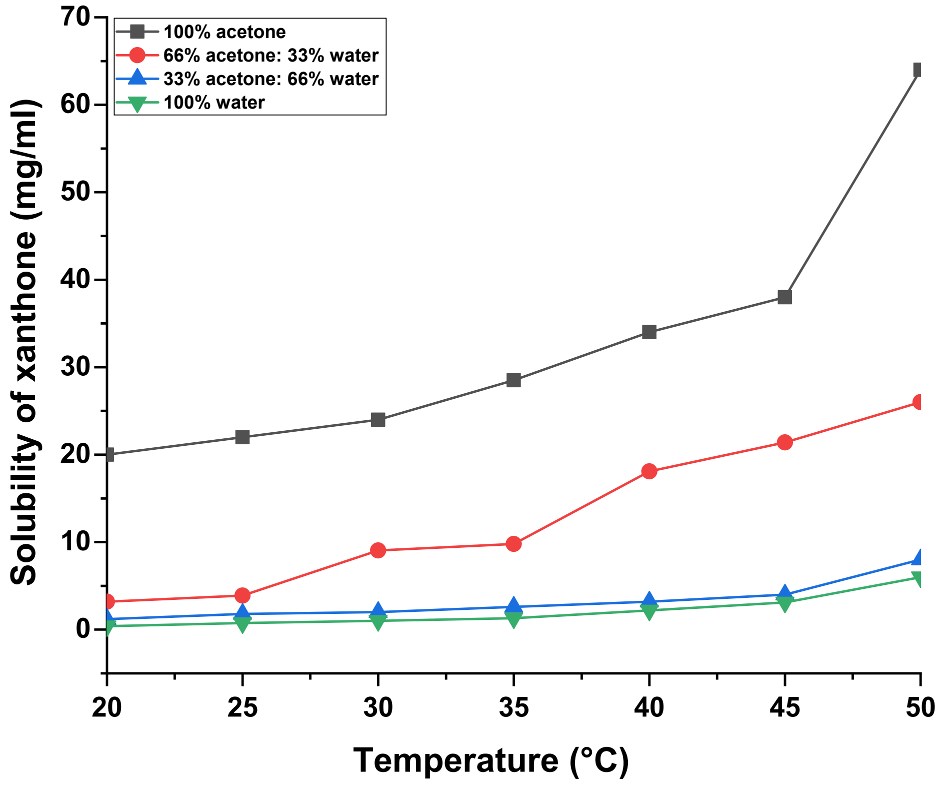

Figure S1: Solubility of xanthone at different temperatures in single solvent and mixed solvent systems.

Table S1: single crystal data and structure refinement for xanthone.

	**jp_xanth_a60w40_twin1_hklf4**
**Crystal data**	
**Chemical formula**	$C_{13}H_8O_2$
**M_r**	196.19
**Crystal system, space group**	Monoclinic, $P2_1$
**Temperature (K)**	130
***a*, *b*, *c* (Å)**	4.8949 (2), 13.4141 (6), 13.9886 (6)
**β (°)**	92.930 (4)
**V (Å^3)**	917.30 (7)
Z	4
***F*(000)**	408
**D_x (Mg m^{-3})**	1.421
**Radiation type**	Cu *K*α
**No. of reflections for cell measurement**	1987
**μ (mm^{-1})**	0.78
**Crystal size (mm)**	0.17 × 0.11 × 0.07
**Data collection**	
**Diffractometer**	SuperNova, Dual, Cu at home/near, Atlas
**Absorption correction**	Gaussian<br>*CrysAlis PRO* 1.171.40.53 (Rigaku Oxford Diffraction, 2019) Numerical absorption correction based on gaussian integration over a multifaceted crystal model Empirical absorption correction using spherical harmonics, implemented in SCALE3 ABSPACK scaling algorithm.
**T_{min}, T_{max}**	0.909, 0.950
**No. of measured, independent and observed [$I > 2\sigma(I)$] reflections**	3654, 3654, 3036
**θ values (°)**	θ_{max} = 73.0, θ_{min} = 4.6
**$(\sin \theta/\lambda)_{max}$ (Å^{-1})**	0.620
**Range of h,k,l**	*h* = −5 5, *k* = −16 16, *l* = −15 17
**Refinement**	
**$R[F^2 > 2\sigma(F^2)]$, $wR(F^2)$, S**	0.031, 0.078, 0.98
**No. of reflections**	3654
**No. of parameters**	272
**No. of restraints**	1
**H-atom treatment**	H-atom parameters constrained
**$\Delta\rho_{max}$, $\Delta\rho_{min}$ (e Å^{-3})**	0.15, −0.16
**Absolute structure**	Classical Flack method preferred over Parsons because s.u. lower.

**Absolute structure parameter**	0.04 (16)

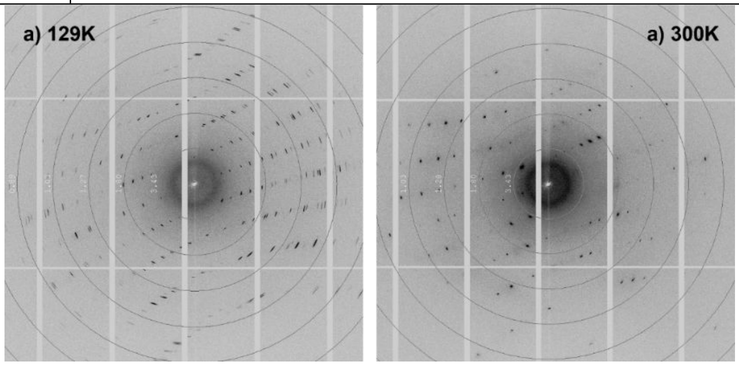

Figure S2: Diffraction images of xanthone crystal at a) 129 K and b) at 300K.

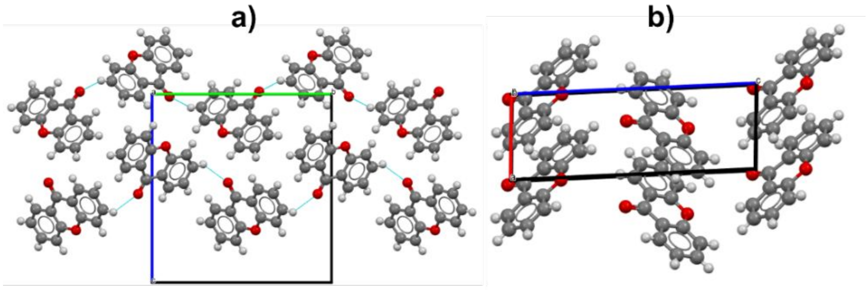

Figure S3: Crystal packing of xanthone. A) view along the *b* axis b) View along the *a* axis. Weak hydrogen bond interactions are represented in blue dashed lines.

Table 2: Crystallographic data comparison between orthorhombic and monoclinic xanthone. The data are taken from the Crystallographic Structure Database (CSD).

	**Xanthone (REFCODE: ZZZTXI4)**[1]	**Xanthone (REFCODE: ZZZTXI3)** [2]
**Chemical formula**	$C_{13}H_8O_2$	$C_{13}H_8O_2$
**Space Group**	Monoclinic, $P2_1$	Orthorhombic, $P2_12_12_1$
**Temperature (K)**	130	298
**a, b, c (Å)**	4.8949 (2), 13.4141 (6), 13.9886 (6)	4.8930 (6), 13.6576 (15), 14.134 (2)
**α, β, γ (°)**	90, 92.930 (4), 90	90, 90, 90
**V (Å^3)**	917.30 (7)	944.5 (2)
**Z**	4	4
**Radiation type**	Cu Kα	Mo Kα
**R[$F^2 > 2\sigma(F^2)$], wR(F^2), S**	0.031, 0.078, 0.98	0.057, 0.097, 1.01

Table 3: Cell length and angle indexation of the SXRD xanthone dataset. Datasets are sorted by increasing temperature.

**Data**	**Temperature (K)**	**a (Å)**	**b (Å)**	**c (Å)**	**α**	**β**	**γ**
Xanthone	129	4.882	13.390	13.917	90.00	92.86	90.00
Xanthone	139	4.092	13.424	14.020	90.00	93.08	90.00
Xanthone	149	4.899	13.456	14.038	90.00	92.89	90.00
Xanthone	159	4.897	13448	14.030	90.00	92.92	90.00
Xanthone	169	4.906	13.462	14.041	90.00	93.05	90.00
Xanthone	179	4.922	13.464	14.041	90.00	92.78	90.00
Xanthone	189	4.906	13.475	14.080	90.00	92.78	90.00
Xanthone	199	4.912	13.494	14.075	90.00	92.82	90.00
Xanthone	209	4.911	13.498	14.065	90.00	92.82	90.00
Xanthone	219	4.908	13.527	14.059	90.00	92.18	90.00
Xanthone	229	4.098	13.540	14.101	90.00	92.51	90.00
Xanthone	239	4.902	13.557	14.104	90.00	92.28	90.00
Xanthone	249	4.903	13.578	14.122	90.00	92.06	90.00
Xanthone	259	4.908	13.600	14.111	90.00	91.70	90.00
Xanthone	269	4.904	13.620	14.134	90.00	91.68	90.00
Xanthone	279	4.923	13.630	14.140	90.00	91.24	90.00
Xanthone	289	4.890	13.650	14.275	90.00	90.84	90.00
Xanthone	300	4.910	13.660	14.250	90.00	90.56	90.00

References

1. Trapp, N., Vastakaite, G., Wennemers, H. (2019) CCDC 1906607: Experimental Crystal Structure Determination DOI: 10.5517/ccdc.csd.cc21zzgh (ZZZTXI14).
2. Jing Tang, Shijun Zhao, Yuanyuan Wei, Zhengjun Quan, Congde Huo, Org.Biomol.Chem. (2017), 15, 1589, doi:1 0.1039/C7OB00080D. (ZZZTXI3)
